# The Connection Between Rap1 and Talin1 in the Activation of Integrins in Blood Cells

**DOI:** 10.3389/fcell.2022.908622

**Published:** 2022-06-01

**Authors:** Hao Sun, Frederic Lagarrigue, Mark H. Ginsberg

**Affiliations:** ^1^ Department of Medicine, University of California San Diego, San Diego, CA, United States; ^2^ Institut de Pharmacologie et Biologie Structurale, Université de Toulouse, CNRS, Université Paul Sabatier, Toulouse, France

**Keywords:** integrin activation, cell adhesion, cell migration, signaling, blood cells

## Abstract

Integrins regulate the adhesion and migration of blood cells to ensure the proper positioning of these cells in the environment. Integrins detect physical and chemical stimuli in the extracellular matrix and regulate signaling pathways in blood cells that mediate their functions. Integrins are usually in a resting state in blood cells until agonist stimulation results in a high-affinity conformation (“integrin activation”), which is central to integrins’ contribution to blood cells’ trafficking and functions. In this review, we summarize the mechanisms of integrin activation in blood cells with a focus on recent advances understanding of mechanisms whereby Rap1 regulates talin1-integrin interaction to trigger integrin activation in lymphocytes, platelets, and neutrophils.

## Introduction

Integrin receptors are heterodimeric α and β cell adhesion molecules that play essential roles in cell-to-cell, cell-to-extracellular matrix, and cell-to-pathogen interactions (Hynes, 2002; Hogg et al., 2011). Integrins regulate signaling pathways in blood cells to mediate cell adhesion, cell migration, cell proliferation and cell differentiation (Schwartz et al., 1995; Hynes, 2002). Blood cells, including leukocytes and platelets, dynamically increase the affinity of integrins for their ligands (activation). This is a central event for many cellular functions (Tadokoro et al., 2003; Shattil et al., 2010). Leukocyte integrins β1, β2, and β7 are essential for innate and adaptive immune responses (Hynes, 2002; Luo et al., 2007; Hogg et al., 2011; Vestweber, 2015); and also contribute to many cardiovascular diseases, including atherosclerosis, thrombosis, stroke and peripheral arterial disease (Clemetson and Clemetson, 1998; Galkina and Ley, 2007; Lal et al., 2007; Lu et al., 2008). When rolling leukocytes are stimulated by chemokines or cytokines, the integrins thus activated interact with their ligands on endothelial cells and induce cell arrest and then extravasation during inflammatory responses (Hynes, 2002; Ley et al., 2007). Likewise, binding of integrin αIIbβ3 to its ligand in a high affinity state is a critical step to enable both stable adhesion to the vessel wall and platelet aggregation for effective hemostasis (Bennett and Vilaire, 1979).

Rap1 GTPases act as a molecular switch that controls talin1-dependent activation (Bos et al., 2001; Bos et al., 2003) of β1 (Tadokoro et al., 2003; Shattil et al., 2010), β2 (Katagiri et al., 2003; Simonson et al., 2006), β3 (Petrich et al., 2007a; Petrich et al., 2007b; Nieswandt et al., 2007; Haling et al., 2011), and β7 (Sun et al., 2014; Sun et al., 2018) and integrins. The Rap1-interacting adaptor molecule (RIAM) is an effector of Rap1 that mediating this function in leukocytes (Klapproth et al., 2015; Su et al., 2015; Lagarrigue et al., 2017; Sun et al., 2021). Recent work indicates that direct Rap1 binding to talin1 plays a vital role in integrin activation in platelets (Bromberger et al., 2018; Lagarrigue et al., 2018; Lagarrigue et al., 2020) and also contributes to integrin activation in leukocytes (Lagarrigue et al., 2020; Bromberger et al., 2021; Lagarrigue et al., 2022).

In this review, we discuss recent insights into the mechanisms of talin1 recruitment to integrins and their subsequent activation in blood cells, focusing on the function of the Rap1-talin1 axis in different cell types and classes of integrins.

## Integrin Activation

Integrin α and β subunits are composed of a large ectodomain, a transmembrane domain (TMD) and a cytoplasmic tail (CT) (Hynes, 1987). The integrin β subunit head contains a cation-dependent ligand binding site (Loftus et al., 1990) that is part of an A domain (sometimes called “I domain’ meaning inserted domain) (Xiong et al., 2001). Four β1 integrins and all four β2 integrins of leukocytes contain a second A domain in the α subunit which serves as the primary ligand binding site (Lee et al., 1995). Conformational changes in the extracellular domain increased the affinity of integrins for monomeric ligands and this results from the destabilization of the association of transmembrane and cytoplasmic *α* and β domains (Hughes et al., 1996; Kim et al., 2003; Mould et al., 2003; Luo et al., 2004; Partridge et al., 2005). Integrins exhibit at least three major conformational states (Takagi et al., 2002; Luo et al., 2007; Chen et al., 2010; Springer and Dustin, 2012): inactive (low affinity, bent-closed, resting state), intermediate (low affinity, extended-closed), and active (high affinity, extended-open). The extended-high affinity state is induced by a conformational rearrangement in the A domain that follows the displacement of the α7 helix in this structure (Emsley et al., 2000). A current model proposes that talin1 associates with the cytoplasmic tail of the β subunit and disrupts the stabilization of the inner membrane clasp (Anthis et al., 2009; Lau et al., 2009). In addition, the binding of talin1 to membrane lipids is also essential for activation (Anthis et al., 2009) because it enables a change in the topology of the β transmembrane domain that disrupts an outer membrane clasp (Kim et al., 2011a) thus breaking the two constraints that stabilize the αβ TMD association. Recent studies with β7 integrin have confirmed the importance of transmission of β TMD topology that disrupts the outer membrane clasp (Sun et al., 2018) *in vivo* in the development of gut-associated lymphoid tissue. Furthermore, studies of β2 integrins have shown that a mutation that reduces transmission changes in TMD topology blocks integrin extension (Sun et al., 2020) but not the high-affinity conformation.

## Integrin Adaptor Proteins

The inside-out signaling of integrins is precisely controlled by the binding of various intracellular adapter proteins to integrins (Abram and Lowell, 2009). A well-described pathway of integrin activation in leukocytes and platelets involves production of Ca^2+^ and diacylglycerol, protein kinase C (PKC) and activation of Rap1 GTPase. Then, Rap1 interacts with its effector Rap1-GTP-interacting-adaptor molecule (RIAM, product of the *APBB1IP* gene), which in turn recruits talin1 to the plasma membrane to facilitate its association with integrin (Han et al., 2006; Lee et al., 2009; Lagarrigue et al., 2016). Kindlin3 also participates in this process. Kindlins play an important role in integrin-mediated functions (Tu et al., 2003; Kloeker et al., 2004; Moser et al., 2008; Moser et al., 2009); however, a major role of kindlins is also to enable clustering of integrins leading to avidity modulation and resultant outside-in signaling (Ye et al., 2013).

Rap1 is a small GTPase essential for inside-out signaling of the integrin (Franke et al., 1997; Jeon et al., 2007). It is activated downstream of many agonists by the action of guanine nucleotide-exchange factors (GEFs), which trigger the switch from GDP-bound Rap1 to GTP-bound Rap1, resulting in activation of integrins in leukocytes or platelets. Several Rap1 GEFs such as diacylglycerol-regulated GEF1 (CalDAG-GEF1) and Ca^2+^ have been identified and are activated in response to a diverse set of upstream stimuli, indicating that multiple pathways may converge on Rap1 GTPases (Abram and Lowell, 2009). RapGEF1, RapGEF3 and RapGEF6 activate Rap1 and promote integrin activation in leukocytes (Abram and Lowell, 2009), while CalDAG-GEF1 is important for Rap1 activation in platelets (Eto et al., 2002; Crittenden et al., 2004; Canault et al., 2014; Lozano et al., 2016). GTP binding causes a conformational change in Rap1 that enables it to engage other proteins that serve as effectors of Rap1-regulated functions.

The ability of intracellular signaling pathways to induce integrin activation depends on the binding of talin1 to the integrin β tail (Tadokoro et al., 2003; Ye et al., 2010; Kim et al., 2011b; Lefort et al., 2012). Talin1 is a major cytoskeletal protein that links integrins and the actin cytoskeleton through its head domain (N-terminal) binding to the integrin β CT domains, and its rod domain (C-terminal) binding to F-actin (Critchley and Gingras, 2008). In mammals, the *Tln1* and *Tln2* genes encode talin1 and talin2 respectively, but only talin1 is expressed in blood cells and endothelium. The talin1 head domain (THD) comprises an atypical FERM (band 4.1, ezrin, radixin, and moesin) domain grouping four subdomains: F1, F2, F3, and an F0 subdomain derived from the F1 duplication (Calderwood et al., 2013). Structural studies have shown that the F3 subdomain interacts with the conserved proximal NPXY motif in the CT domain of β integrin, leading to the conformational change of integrin and triggering integrin activation (Hemmings et al., 1996; Garcia-Alvarez et al., 2003; Wegener et al., 2007; Shattil et al., 2010; Kim et al., 2011b). The rod domain has 13 subdomains (R1-R13), which include binding sites for RIAM, integrins, vinculin and F-actin (Hemmings et al., 1996; Goult et al., 2013). The mechanism of talin1 activation is still controversial. Binding to PIP5Kγ facilitates the recruitment of talin1 to the plasma membrane, where the talin1-F2 and -F3 subdomains interact with phosphatidylinositol-4,5-bisphosphate (PI(4,5)P_2_). This binding disrupts the association between the talin1-head domain with the tail and exposes integrin binding sites (Di Paolo et al., 2002; Ling et al., 2002). Germline deletion of talin1 results in embryonic lethality (Monkley et al., 2000), The consequences of the loss of talin1 in mouse blood cells by conditional inactivation have explicitly shown the key role of talin1 in the activation of integrins in leukocytes and platelets (Petrich et al., 2007b; Nieswandt et al., 2007; Manevich-Mendelson et al., 2010; Lefort et al., 2012; Klann et al., 2017). Although the mechanism by which talin1 activates integrins has been well described, there is a need to understand precisely the details of signal transduction events from cell stimulation to the recruitment of talin1 to integrins.

## The Rap1-RIAM-talin1 Axis vs. the Rap1-Talin1 Axis

Rap1 is an important signaling hub for the integration of adhesive signals. Rap1 exists in two isoforms, Rap1a and Rap1b, which are encoded by two different genes. RIAM is an effector of Rap1 that binds talin1 to allow its interaction with the cytoplasmic tails of integrins (Lafuente et al., 2004; Lee et al., 2009; Chen et al., 2017). RIAM belongs to the family of Mig-10/RIAM/Lamellipodin (MRL) adapter proteins which are characterized by a structural module comprising a Ras association domain (RA) and a pleckstrin homology domain (PH). RIAM forms an autoinhibitory conformation by an intramolecular interaction between the inhibitory (IN) segment and the RA domain at its binding site to Rap1 and on which the packing of two RIAM molecules by the intermolecular interaction of the PH domain masks the binding to phosphoinositides (Chang et al., 2019; Cho et al., 2021). Phosphorylation of the IN segment and PH domain by focal adhesion kinase (FAK) and proto-oncogene tyrosine-protein kinase (Src), respectively, releases these inhibitory conformations and contribute to RIAM activation thus facilitating its interaction with Rap1 and PI(4,5)P_2_ to promote talin1-dependent integrin activation (Chang et al., 2019; Cho et al., 2021).

RIAM is crucial for Rap1-dependent integrin activation in leukocytes (Klapproth et al., 2015; Su et al., 2015; Lagarrigue et al., 2017; Sun et al., 2021). RIAM is mainly expressed in leukocytes where it regulates the function of β2 integrins to support their adhesion and migration (Klapproth et al., 2015; Su et al., 2015). In contrast, RIAM is dispensable for α4β1 integrin activation and functions (Klapproth et al., 2015; Su et al., 2015). RIAM depletion in CD4^+^ T cells inhibits antigen-dependent autoimmunity by interfering with the interaction between effector T cells and antigen presenting cells (Lagarrigue et al., 2017). Interestingly, RIAM has been shown to interact with kindlin-3 even before it binds talin1 (Kliche et al., 2012), but whether RIAM directly interacts with kindlin-3 is unknown. Recently, we showed that RIAM is required for integrin activation in conventional T (Tconv) cells but is dispensable in regulatory T (Treg) cells, although Rap1 is necessary for Treg cell function and RIAM is expressed in Treg cells (Sun et al., 2021).

Lamellipodin (Lpd) (Krause et al., 2004) is a paralogue of RIAM (Lafuente et al., 2004) that compensates for the deficiency of RIAM in the activation of integrins in Treg cells (Sun et al., 2021). Similar to RIAM, Lpd contains talin1 binding sites and triggers integrin activation (Watanabe et al., 2008; Lee et al., 2009). In addition to regulating talin1-dependent integrin activation, RIAM also contributes to β2 integrin-mediated outside-in signaling in neutrophils and macrophages (Klapproth et al., 2015; Torres-Gomez et al., 2020). On the other hand, the role of Lpd in the outside-in signaling of integrins is poorly understood and deserves to be explored.

*In vivo* studies in mice have revealed that loss of RIAM inhibits integrin activation in a cell type-specific manner. RIAM loss can ameliorate autoimmune diseases such as experimental type I diabetes (Lagarrigue et al., 2017) or inflammatory bowel disease (Sun et al., 2021) by suppressing integrin-mediated activation of Tconv cells while retaining Treg cell function. Platelets express little RIAM or Lpd (Klapproth et al., 2015). Mice deficient in Lpd and RIAM exhibit intact platelet integrin activation (Klapproth et al., 2015; Lagarrigue et al., 2015; Stritt et al., 2015; Su et al., 2015; Sun et al., 2021) and normal hemostasis. Moreover, the fact that RIAM deficiency affects leukocyte integrin β2 function less than talin1 deficiency suggests the existence of alternative RIAM-independent pathways that regulate the recruitment of talin1 to integrins (Klapproth et al., 2015; Sun et al., 2021).

NMR studies indicated a direct interaction between Rap1b and the F0 subdomain on talin1 with low affinity (Goult et al., 2010), suggesting that it is a mechanism of recruitment of talin1 to integrins (Zhu et al., 2017). The direct interaction between TalinB and Rap1 is required for adhesion of Dictyostelium cells (Plak et al., 2016). A mutation that blocks the binding of Rap1 to the F0 subdomain of talin1 is an embryonic lethal mutation in Drosophila (Camp et al., 2018). A quantitative study of the proteome in murine platelets revealed that Rap1 and talin1 are highly expressed in murine platelets at an equimolar ratio (Zeiler et al., 2014). In the absence of other known effectors of Rap1 in platelets, it became clear that the direct interaction of Rap1 with talin1 F0 could play a vital role in integrin activation in platelets. However, a talin1 (R35E) mutation in the F0 subdomain, which blocks direct binding of Rap1, has a modest effect on talin1 induced αIIbβ3 activation (Lagarrigue et al., 2018). In parallel, Moser’s group generated a Tln1^3mut^ mouse carrying the K15A, R30A, R35A mutations in the F0 subdomain of talin1 and showed a weak contribution of the F0-Rap1 interaction in the activation of αIIbβ3. Talin1 (R35E) and Tln1^3mut^ mice are apparently healthy, fertile, and viable, and showed bleeding times similar to wild-type littermates, indicating mild defects in platelet aggregation and hemostasis (Bromberger et al., 2018; Lagarrigue et al., 2018). Thus, Rap1-talin1 F0 binding has a minor effect on platelet integrin activation despite a critical role of Rap1 and talin1 for αIIbβ3 activation and platelet function (Petrich et al., 2007b; Nieswandt et al., 2007; Stefanini et al., 2018). We recently found a new Rap1 binding site in the talin1 F1 domain (Gingras et al., 2019). The talin1 (R118E) mutation in the F1 domain profoundly reduces the ability of F1 to bind Rap1 and significantly disrupts the ability of Rap1 to mediate talin1-induced integrin activation in platelets (Gingras et al., 2019; Lagarrigue et al., 2020). The talin1 F1 subdomain plays a distinct and much more central role than the F0 domain in Rap1-mediated integrin activation in mammalian cells. Moreover, loss of both Rap1 binding sites in talin1 (R35E,R118E) mutant mice causes a much greater defect in platelet integrin activation, similar to that caused by knockout of both Rap1 a and b isoforms (Stefanini et al., 2018; Lagarrigue et al., 2020). These results suggest that Rap1 binding to the F0 and F1 subdomains of talin1 induces talin1-dependent integrin activation in platelets and plays a fundamental role in hemostasis and thrombosis.

We now have a more complete picture of how the talin1 head domain interacts with the membrane. Positively charged patches in the talin1 F2 and F3 areas bind to membranes to stabilize the weak interaction of talin1 F3 with the cytoplasmic β integrin domain to explain the membrane dependency of talin1-induced activation (Ye et al., 2010; Chinthalapudi et al., 2018; Bromberger et al., 2019). Additionally, the Rap1 binding site in the F1 subdomain of talin1 functions in conjunction with a unique inserted loop that interacts with membrane lipids to enable Rap1 to contribute significantly to the membrane targeting of talin1 (Goult et al., 2010; Gingras et al., 2019). The proximity of the putative membrane-binding helix of the F1 loop to the F1-linked geranyl-geranyl fragment of Rap1 provides a compelling model to explain the complementary role of these two membrane-binding sites in the activation of the integrin. Furthermore, while all studies have focused only on the binding of Rap1b to talin1 (Goult et al., 2010; Bromberger et al., 2018; Lagarrigue et al., 2018; Gingras et al., 2019), we can anticipate that Rap1a may also interact directly with talin1 given that Rap1a and Rap1b share 95% sequence identity. Moreover, the main positively charged residues in talin1 for Rap1 binding are conserved in talin2, in particular K15 and R35 in talin1 F0 and R98 and R118 in talin1 F1, suggesting that Rap1 could also interact directly with talin2. Future studies are needed to determine if direct binding can be extended to each of the Rap1 and talin isoforms.

## Cell-Type Specific Connections Between Rap1 and Talin1

**FIGURE 1:**
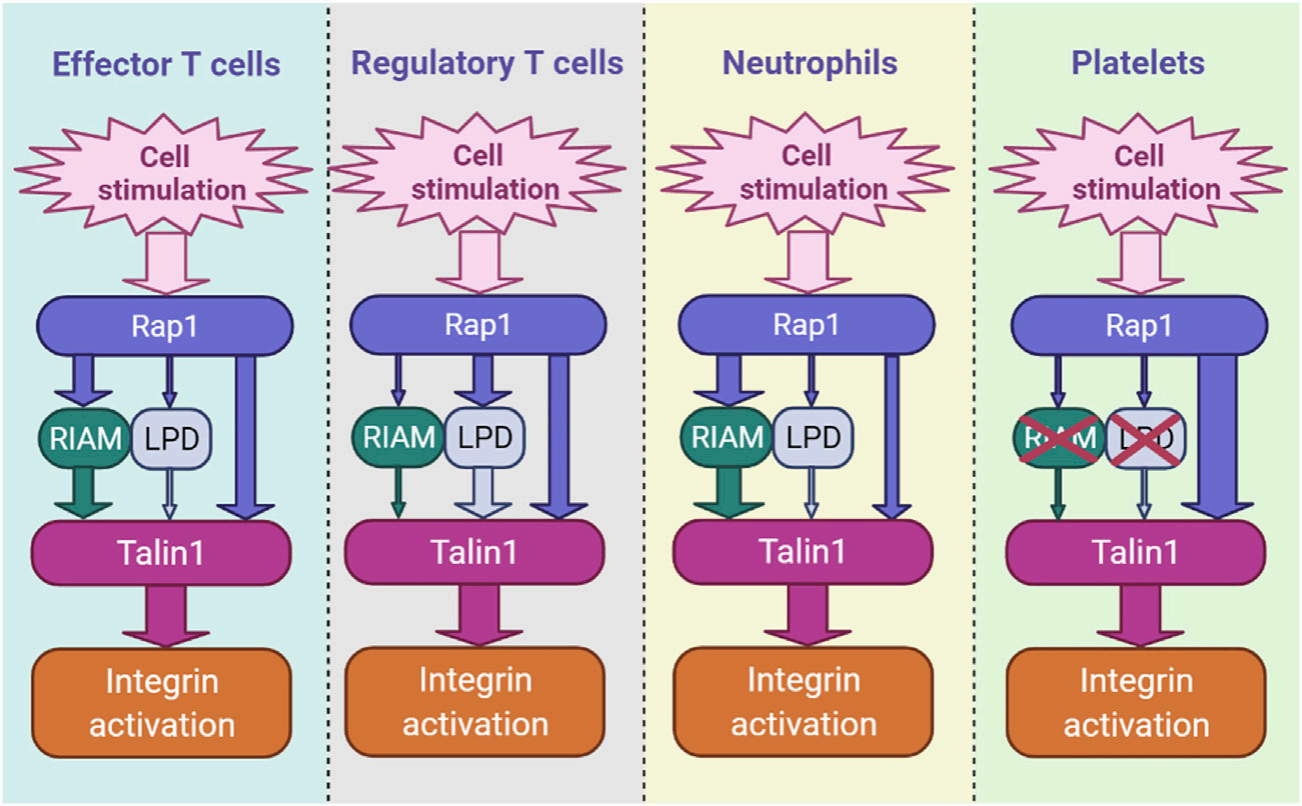
The hierarchy of integrin activation pathways differs by cell type. RIAM- and LPD-dependent pathways and the direct binding of Rap1 to talin1 coexist and contribute in parallel in blood cells, including immune cells. The thickness of the arrow indicates the relative importance of each channel.

The mechanism of recruitment of talin1 by Rap1 has been the subject of several recent studies (Zhu et al., 2017; Bromberger et al., 2018; Bromberger et al., 2019; Gingras et al., 2019; Lagarrigue et al., 2020; Bromberger et al., 2021; Lagarrigue et al., 2022). Interestingly, this dialogue varies by cell type and integrin class. In platelets, lacking endogenous RIAM, talin1 (R35E, R118E) mutations profoundly inhibit integrin αIIbβ3 activation and platelet aggregation, revealing that talin1 is the sole effector of Rap1 (Lagarrigue et al., 2020) (Figure 1).

We have also recently shown that direct binding of Rap1 to talin1 regulates integrin activation in Tconv and Treg cells (Lagarrigue et al., 2022). Inhibition of Rap1 binding to talin1 (R35E,R118E) induces a significant decrease in the activation of integrins α4β1, α4β7 and αLβ2 in Tconv and Treg cells, resulting in defects in T cell homing and functions. However, unlike platelets, RIAM is required for optimal integrin activation in T cells (Klapproth et al., 2015; Su et al., 2015; Lagarrigue et al., 2017; Sun et al., 2021) (Figure 1). While the loss of RIAM strongly impacts the activation of integrins β2 and β3, the effect is minimal on integrin α4β1. In contrast, T cells expressing talin1 (R35E,R118E) show only partial loss of integrin activation (Lagarrigue et al., 2022). RIAM-deficient T cells expressing talin1 (R35E,R118E) show a much deeper impairment in αLβ2, α4β1 and α4β7 activation. RIAM and Lpd depletion combined with talin1 (R35E,R118E) further significantly abrogates integrin activation in Treg cells. RIAM overexpression circumvents the integrin activation deficit in talin1 (R35E,R118E)-expressing T cells, suggesting that RIAM may compensate for Rap1 binding to talin1 during integrin activation (Lagarrigue et al., 2022). Altogether, these results indicate the importance of the interaction between Rap1 and talin1 which promote the activation of integrins αLβ2, α4β1 and α4β7 in T cells (Figure 1).

The two axes Rap1-talin1 and Rap1-RIAM-talin1 act synergistically to regulate integrin β2 activation in leukocytes (Bromberger et al., 2021; Lagarrigue et al., 2022). On the other hand, what distinguishes these two paths is not so clear. The involvement of RIAM adds a higher level of regulation since RIAM must be phosphorylated by Fak and Src to be activated (Chang et al., 2019; Cho et al., 2021). Moreover, RIAM, but also Lpd, couple talin1-mediated integrin activation to the regulation of actin dynamics by forming a molecular complex that results in cellular protrusions at the tips of migrating cells (Lagarrigue et al., 2015). It is therefore tempting to see in the expression of MRL proteins in leukocytes a reflection of their migratory capacity which requires fine tuning of the activation of integrins and the regulation of the actin cytoskeleton. In contrast, one might speculate that in platelets, which do not express RIAM and are much less motile, the Rap1-talin1 pathway occurs more rapidly due to the extra time required for RIAM or Lpd to be phosphorylated and released from the autoinhibition in leukocytes. Further research is needed to determine whether direct binding of Rap1 to talin1, without the use of MRL proteins as an intermediary, reduces the time required for integrin activation in platelets to specifically mediate their binding to the vascular wall under a strong shear force.

Neutrophils are generally considered to be the first immune cells to defend against pathogens or infections. Aberrant neutrophil infiltration is seen in patients with autoimmune disease (Caradonna et al., 2000; Smith et al., 2005). Loss of Rap1 can markedly suppress neutrophil functions largely because it inhibits integrin activation (Bromberger et al., 2018). The Tln1^3mut^ mouse (Bromberger et al., 2018), like the talin1 (R35E) mutant mouse (Lagarrigue et al., 2018), does not show leukocytosis, and the neutrophils of the Tln1^3mut^ mice show a slight adhesion defect and a reduced extravasation. However, Tln1^3mut^ mutation combined with the loss of RIAM clearly suppresses neutrophil integrin functions (Bromberger et al., 2021). In contrast, *Tln1* ^*R118E/R118E*^ mice exhibit leukocytosis (Lagarrigue et al., 2020), which affects both neutrophils and lymphocytes, suggesting that the F1-Rap1 interaction has a greater functional impact than the F0-Rap1 interaction. The role of Rap1-talin1 binding in neutrophil adhesion and arrest deserves further investigation.

## Conclusion

Binding of talin1 to integrin is a terminal event in the inside-out signaling of integrins. Rap1 serves as a convergence point for many adhesive signals. The link between Rap1 and talin1 in integrin activation has long remained a significant gap in our understanding of this event. However, recent advances, partly accelerated by the RIAM and Lpd knockout mice, developed respectively by the laboratories of F.B. Gertler (Klapproth et al., 2015; Su et al., 2015) and M. Krause (Law et al., 2013), have enabled dissections of the role of MRL proteins in different cells. In particular, RIAM appears to play the most important role in most lymphocytes and neutrophils. In Treg cells, however, Lpd is clearly more important. The third connection, via direct binding to Rap1 to two binding sites in the head domain of talin1 (Gingras et al., 2019), is important in all blood cells. In platelets, which express negligible amounts of the MRL proteins, direct binding of Rap1 to talin1 accounts for Rap1-dependent integrin activation (Lagarrigue et al., 2020). Thus, these three pathways act in parallel to connect Rap1 to talin1. The relative contribution of each pathway varies by cell type and integrin type and this variation offers the potential to selectively manipulate integrin activation in a cell type-specific manner.
